# Toxicity and Biological Effects of *Beauveria brongniartii* Fe^0^ Nanoparticles against *Spodoptera litura* (Fabricius)

**DOI:** 10.3390/insects11120895

**Published:** 2020-12-21

**Authors:** Jing Xu, Kaihui Zhang, Andrew G. S. Cuthbertson, Cailian Du, Shaukat Ali

**Affiliations:** 1 Key Laboratory of Bio-Pesticide Innovation and Application, Guangzhou 510642, China; zhibaoxujing@stu.scau.edu.cn (J.X.); alientomologist@gmail.com (K.Z.); ducailian@stu.scau.edu.cn (C.D.); 2 Engineering Research Center of Biological Control, Ministry of Education and Guangdong Province, South China Agricultural University, Guangzhou 510642, China; 3 Independent Science Advisor, York YO10 5AQ, UK; andrew_cuthbertson@live.co.uk

**Keywords:** biological control, entomopathogenic fungus, feeding, growth, toxicity

## Abstract

**Simple Summary:**

Metal-based nanoparticles of different microbial pest control agents have been effective against several pests. This study reports the synthesis of *Beauveria brongniartii* based Fe^0^ nanoparticles (Fe^0^NPs) and their bio-efficacy against *Spodoptera litura* that was observed during this study. The median lethal concentration (LC_50_) of Fe^0^NPs against *S. litura* after 7 days was 59 ppm, whereas the median survival time (LT_50_) for 500 ppm concentrations of Fe^0^NPs was 2.93 days. *B. brongniartii* Fe^0^NPs caused a significant reduction in feeding and growth parameters as well as detoxifying enzyme production by *S. litura* at the end of the experimental period. These findings suggest that *B. brongniartii* Fe^0^NPs can potentially be used in environmentally friendly *S. litura* management programs.

**Abstract:**

Nanotechnology has clear potential in the development of innovative insecticidal products for the biorational management of major insect pests. Metal-based nanoparticles of different microbial pest control agents have been effective against several pests. Synthesis of *Beauveria brongniartii* based Fe^0^ nanoparticles (Fe^0^NPs) and their bio-efficacy against *Spodoptera litura* was observed during this study. *Beauveria brongniartii* conidia were coated with Fe^0^NPs and characterized by applying a selection of different analytical techniques. Ultraviolet (UV) spectroscopy showed the characteristic band of surface plasmon at 430 nm; Scanning electron microscopy (SEM) images showed spherical shaped nanoparticles with a size ranging between 0.41 to 0.80 µm; Energy-dispersive X-ray (EDX) spectral analysis revealed characteristic Fe peaks at 6.5 and 7.1 Kev; the X-ray diffractogram showed three strong peaks at 2*θ* values of 45.72°, 64.47°, and 84.05°. The bioassay studies demonstrated that mortality of 2nd instar *S. litura* larvae following Fe^0^NPs treatment increased with increasing concentrations of Fe^0^NPs at different time intervals. The median lethal concentration (LC_50_) values of Fe^0^NPs against *S. litura* after seven days of fungal treatment was 59 ppm, whereas median survival time (LT_50_) values for 200 and 500 ppm concentrations of Fe^0^NPs against *S. litura* seven days post-treatment were 5.1 and 2.29 days, respectively. *Beauveria brongniartii*-Fe^0^NPs caused significant reductions in feeding and growth parameters (relative growth rate, relative consumption rate, and efficiency of conversion of ingested food) of *S. litura*. *Beauveria brongniartii* Fe^0^NPs induced reduction in glutathione-S-transferase activities throughout the infection period whereas activities of antioxidant enzymes decreased during later periods of infection. These findings suggest that *B. brongniartii* Fe^0^NPs can potentially be used in biorational *S. litura* management programs.

## 1. Introduction

*Spodoptera litura* (Lepidoptera; Noctuidae) is an important cosmopolitan pest with a wide range of hosts, including different vegetable and field crops such as cotton, groundnut, soybean, tomato, sweet potato, and tobacco [1,2]. There are numerous reports of the development of resistance in *S. litura* against a wide range of insecticides, resulting in many sporadic outbreaks of the pests which have led to the failure of crops [3]. Since reports of the presence of this pest in the Hunan province of China, the damage has increased continually. Currently, local farmers mainly depend on synthetic pesticides to control the pest. However, the mismanagement and over-application of synthetic chemicals have caused a high level of resistance to appear in several invertebrate pest populations including *S. litura* [4,5].

Entomopathogenic fungi have been suggested as potential agents for the biological control of different insects for over a century. Under favorable conditions, fungi occur frequently and often cause a significant reduction in insect pest populations [6]. There are several advantages of using entomopathogenic fungi: they have broad-spectrum insecticidal activity, a diversified species range, have complex metabolic types, and offer appropriate safety levels for humans and other non-target organisms [7]. They are also easy to mass-produce and the development of host resistance against them is unlikely to occur. However, one of the most widely used fungi as a biological pesticide, *Beauveria brongniartii,* can be affected by the environment. New agents with better efficacy, durability, and stability therefore need to be developed.

Nanotechnology is an emerging science that will potentially significantly revolutionize the agricultural and food industry by using nanoparticles and nanomaterials for disease and pest control [8]. Nanoparticles can be used in different ways and could be useful for pest control as the mixed formulation of metal and various other materials have already been reported effective against pests [9]. Nanoparticles of silver using different entomopathogenic fungi (*Metarhizium anisopliae*, *Beauveria bassiana,* and *Isaria fumosorosea*) have been reported as effective biopesticides against mosquitoes [10,11,12]. Wang et al. [13] studied the synthesis, characterization, and demonstrated the toxicity of *I. fumosorosea* based zero-valent iron nanoparticles against the sweet potato whitefly, *Bemisia tabaci*.

In this context, the current investigations were carried out to synthesize and characterize *Beauveria brongniartii* Fe^0^ nanoparticles in conjunction with studies on their bio-efficacy against *S. litura*. The main objectives of the current work were: (a) to synthesize and characterize *Beauveria brongniartii* Fe^0^ nanoparticles through different standardized analytical techniques (scanning electron microscopy, energy-dispersive X-ray spectroscopy, X-ray diffractometry, Fourier transmission infrared spectroscopy); (b) to study the concentration mortality responses of *S. litura* to Fe^0^NPs; (c) to measure the influence of Fe^0^NPs on feeding and growth parameters of *S. litura*, and; (d) to measure the effects of Fe^0^NPs on activities of detoxifying enzymes produced by *S. litura.*

## 2. Materials and Methods

### 2.1. Insect Cultures

*Spodoptera litura* individuals, used during these experiments, were collected from experimental fields of South China Agricultural University from cotton plants and reared for multiple generations on a semi-synthetic diet under insecticide-free conditions as outlined by David et al. [14] at the Engineering Research Centre of Biological Control, Ministry of Education, South China Agricultural University.

### 2.2. Fungal Inoculum

*Beauveria brongniartii* strain SB010 (obtained from the Key Laboratory of Biopesticides Innovation and Application of Guangdong Province, South China Agricultural University, Guangzhou, P.R. China) was cultured on Potato Dextrose agar (PDA) plates following the method of Ali et al. [15]. The basal fungal suspension (1 × 10^8^ conidia/mL) to be used during the current experiments was prepared using the method of Ali et al. [16].

### 2.3. Preparation of Fe^0^NPs

*Beauveria brongniartii* Fe^0^ nanoparticles were synthesized extracellularly following the methods of Amerasan et al. [12] and Wang et al. [13]. Five milliliters of fungal suspension (1 × 10^8^ conidia/mL) were inoculated in Erlenmeyer flasks (150 mL) containing freshly sterilized PDA broth. The flasks were placed in a rotary shaker at 150 revolutions per minute (rpm), 26 ± 2 °C for 72 h. Following 72 h, fungal biomass was separated from the broth via filtration across Whatman filter paper No. 01 and washed three times with ddH_2_O to remove any debris. Ten grams of fungal mycelia were then inoculated into the sterilized ddH_2_O (100 mL) and then incubated again on the rotary shaker at 150 rpm, 26 ± 2 °C for 72 h. The fungal broth was again filtered after 72 h and 100 mg zero-valent iron (particle size ≤ 100 nm) was added to the culture filtrates and again incubated at 120 rpm, 26 ± 2 °C for 72 h. The extra-cellularly prepared culture was stored in a refrigerator until required.

### 2.4. Characterization of Fe^0^NPs

The extracellular synthesis of Fe^0^NPs was characterized by applying different analytical techniques used for nanoparticle characterization in previous studies [12,13]. To perform scanning electron microscopy, Fe^0^NPs were processed and fixed by following the method of Wang et al. [13]. The images were captured under SU8010 (Hitachi Ltd, Hitachi, Japan) scanning electron microscope operating at an accelerated voltage of 5.0 kV. The UV-spectroscopy of Fe^0^NPs was performed at different wavelengths (300, 400, 500, 600 nm) in a Nanodrop one spectrophotometer (Thermo Scientific, USA) after 48 and 72 h. Energy-dispersive X-ray spectroscopy (EDX) was performed for further characterization of structure, as well as the composition of Fe^0^NPs. X-ray diffractometry (XRD) analysis was undertaken (using Co-Kα radiation in a Bruker D8 diffractometer, Karlshure, Germany) for the calculation of the crystalline structure of Fe^0^NPs. The Fe^0^NPs were characterized qualitatively through Fourier transformation infrared spectroscopy (FTIR) using a MIR8035 FTIR spectrometer (Thermo Fisher, Bemen, Germany). All the analytical studies were performed on three occasions with fresh samples.

### 2.5. Concentration Mortality Responses of Spodoptera litura to B. brongniartii Fe^0^NPs

The concentration mortality responses of *S. litura* to Fe^0^NPs were studied by following the method of Wu et al. [17]. Briefly, seven different concentrations of Fe^0^NPs, named as T1–T7 in Table 1 were added to an artificial diet before the diet has solidified. The artificial diet was treated with 1 × 10^8^ conidia/mL *B. brongniartii* (T8) to compare the efficacy of Fe^0^NPs with fungal conidia alone while the artificial diet acted as a negative control (T9). The prepared diets were allowed to cool and then maintained at 4 °C until further use. Newly molted *S. litura* larvae (2nd instar) were starved for 3 h. Each larva was transferred to a small plastic cup (4 cm diameter) where they were individually fed on a treated or control diet (1 g). There were five replicates of each treatment and every replicate contained thirty larvae within each treatment. The plastic cups having insects belonging to different treatments were placed in an incubator (25 ± 2 °C; 80 ± 5% relative humidity and 16 h:8 h (light:dark) photoperiod). A freshly treated diet was placed in each plastic cup daily and the mortality of larvae was recorded until 7 days post-treatment.

### 2.6. Influence of B. brongniartii Fe^0^NPs on Feeding and Growth of Spodoptera litura

The changes in the feeding and growth parameters of *S. litura* were measured to determine the behavioral changes in response to Fe^0^NPs treatment. The 3rd instar *S. litura* (10 individuals) were fed on an artificial diet (1 g) treated with different treatments as shown in Table 1. The larvae (10 individuals) were individually placed in plastic cups (diameter: 3 cm, height: 4 cm) to feed on 1 g of pre-treated as well as the control diet. The experimental setup was incubated at 25 ± 2 °C, 80 ± 5% relative humidity, and 16 h:8 h (light:dark) photoperiod. The whole experimental setup was performed on three occasions. The larvae and artificial diet were weighed every day by using an electronic balance (precision: 0.0001 g) to record the larval weight gain and food weight loss until 15 days post-application on daily basis. The weight of the 3rd instar larvae at 12 h post-molting was used as the starting weight.

The feeding and larval growth data were used to calculate relative growth rate per unit weight of insect (RGR), relative consumption rate (RCR), and efficiency of conversion of ingested food (ECI); all of which were calculated through the following equations [18,19].

RGR = Change in larval weight per day/initial larval weight

RCR = Change in weight of larval diet per day/initial weight of larval diet

ECI = (Weight gained by insects per day)/(weight of food consumed per day) × 100

### 2.7. Effects of B. brongniartii Fe^0^NPs on Activities of Detoxifying Enzymes

Newly molted 4th instar *S. litura* larvae were fed on an artificial diet treated with one of four different treatments (Fe^0^NPs: 250 ppm; Fe^0^NPs: 500 ppm; *B. brongniartii* conidial suspension: 1 × 10^8^ conidia/mL and ddH_2_O as control) for enzymatic studies. The treated larvae were placed in plastic cups followed by incubation at 26 ± 2 °C; 65 ± 5% relative humidity. Following 3, 5, and 7 days, the samples (two larvae) were collected and dissected to get access to their fat bodies [19,20]. Each treatment was performed three times and each replicate consisted of twenty larvae.

Fat bodies were homogenized in phosphate buffer pH 7.3 (Nanjing Jiancheng Bioengineering, nanjing, China) (SOD, CAT and POD assay) and pH 7.5 (Nanjing Jiancheng Bioengineering, nanjing, China) (GST assay) at 4 °C followed by centrifugation at 10,000 *g* for 10 min at 4 °C The supernatant was retained for enzyme assays.

The total protein content of the supernatant was determined by Bradford assay, using bovine serum albumin (BSA) as standard [21].

The total superoxide dismutase (SOD) activity was analyzed following Beauchamp and Fridovich [22] through nitroblue tetrazolium reduction. The unit of SOD activity was described as the quantity of SOD needed to inhibit nitroblue tetrazolium reduction by 50%.

The method of Beers and Sizer [23] was adopted to analyze total catalase (CAT) activity. The decomposition of hydrogen peroxide was observed at 240 nm and one unit of enzyme activity was defined as the quantity of enzyme which can decompose 1 mM H_2_O_2_/min at an initial H_2_O_2_ concentration of 30 mM at 25 °C and pH 7.0.

The peroxidase (POD) activity was quantified through the method of Simon et al. [24] by observing changes in absorbance at 420 nm. Enzyme activity was expressed as units per mg protein (U/mg protein).

The glutathione S- transferase (GSTs) was conducted using the procedures developed by Habig and Jakoby [25]. Incubation was carried out at 25 °C for 5 min in 0.1 M Na-phosphate buffer (pH 6.5) containing 1 mM glutathione, 1 mM dinitrochilorobenzene, and 20 μL of the sample. The reaction was initiated by adding dinitrochilorobenzene solution in acetone. The concentration of 5-(2,4-dinitrophenyl) glutathione produced during the reaction was measured spectrophotometrically at a wavelength of 340 nm. One unit of enzyme will conjugate 10.0 nM of 1-Chloro-2,4-dinitrobenzene with reduced glutathione per minute.

### 2.8. Data Analysis

A percentage of insect mortality was subjected to probit analysis to calculate median lethal concentration and median lethal time values. The remaining data were analyzed by analysis of variance and significant differences between means were calculated by Tukey’s HSD test (*p* ≤ 0.05). All data analyses were performed by using SAS 8.1 software [26].

## 3. Results

### 3.1. Characterization of B. brongniartii Fe^0^ Nanoparticles

The supplementation of Fe^0^ into conidial filtrate of *B. brongniartii* induced a reduction process resulting in a change of the suspension’s color (black to dark grey) at 3 days post-incubation which confirmed the formation of Fe^0^NPs (Supplementary Figure S1).

**Figure 1:**
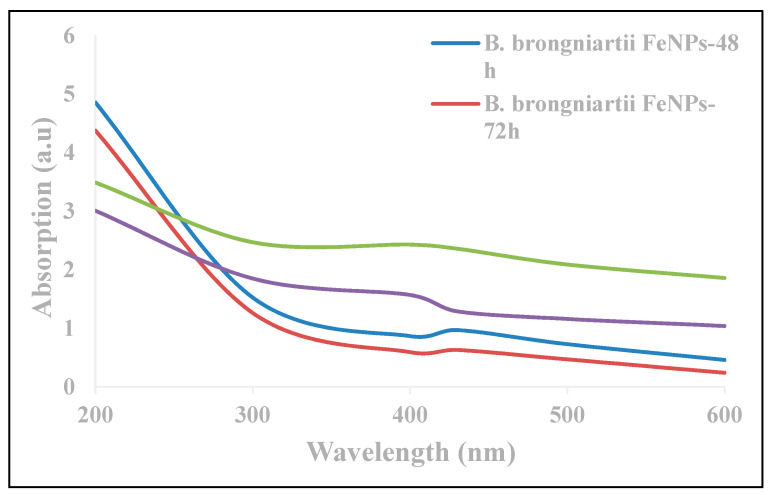
Absorption spectra of *Beauveria brongniartii* Fe^0^ nanoparticles and *Beauveria brongniartii* conidia at different wavelengths after 48 and 72 h.

The UV analysis of aqueous suspension further confirmed the synthesis of Fe^0^NPs. The UV-absorption spectra of samples obtained at different time intervals showed a consistent decrease in UV spectra at different wavelengths with a specific surface plasmon absorption band between 400–450 nm (Figure 1).

**Figure 2:**
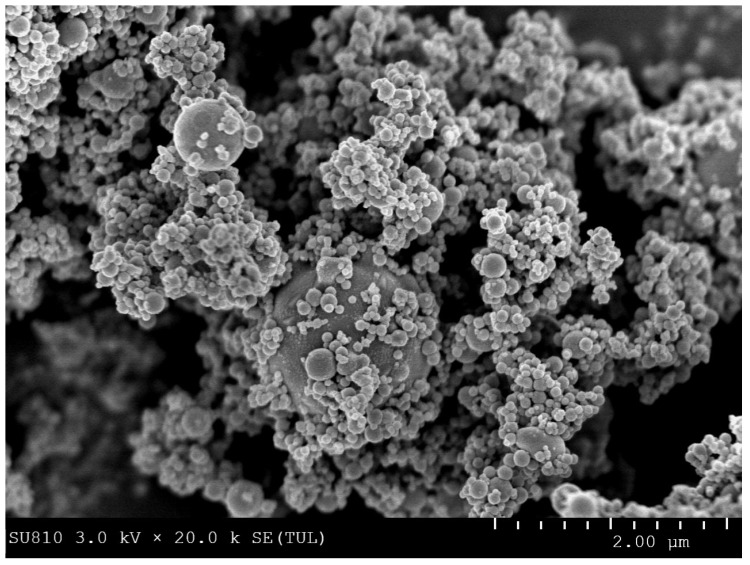
Scanning electron micrographs of *Beauveria brongniartii* Fe^0^NPs.

The SEM images revealed adherence of Fe^0^ particles onto the *B. brongniartii* conidial surface indicating the proper formation of Fe^0^NPs (Figure 2).

The EDX spectral analysis revealed characteristic Fe peaks at 6.5 and 7.1 Kev along with a signal of Mn (Supplementary Figure S2).

**Figure 3:**
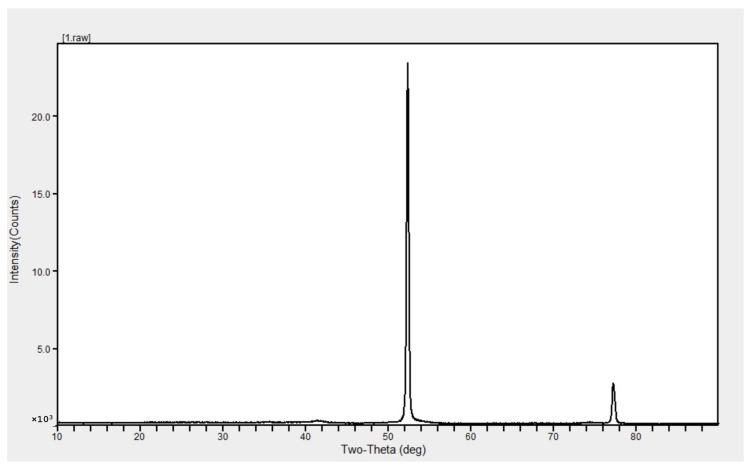
X-ray diffraction pattern of *Beauveria brongniartii* Fe^0^NPs.

X-ray diffractometry (XRD) analysis of Fe^0^NPs showed diffracted intensities at 2*θ* angles ranging from 10° to 90°. The target of XRD analysis was CoKα with a wavelength of 1.743 Å. The X-ray diffractogram showed two strong peaks at 2*θ* values of 52.42° and 77.25° (Figure 3).

**Figure 4:**
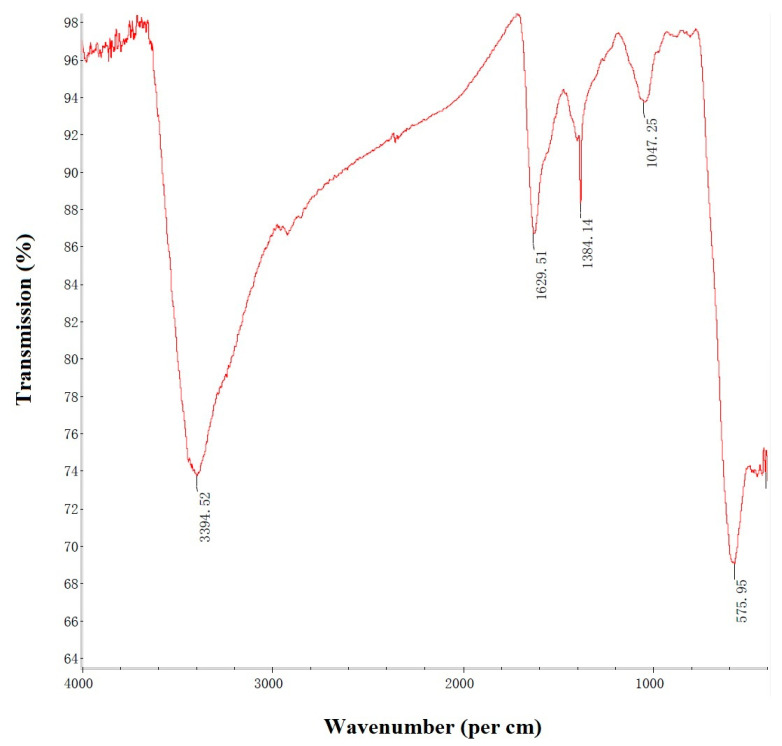
Fourier-transform infrared spectroscopy pattern of *Beauveria brongniartii* Fe^0^NPs.

The FTIR spectroscopic analysis was carried out for the identification of biomolecules responsible for the reduction of Fe^0^ ions as well as the capping of bio-reduced Fe^0^NPs. The FTIR spectra of Fe^0^NPs showed absorption beaks at 3394.52, 1629.51, 1384.41, 1047.25, and 575.95 in the region of 4000–400 cm^−1^ (Figure 4). The analysis of spectral beaks showed the existence of O-H giving a very strong and broad beak, C≡C showed a medium-strong beak, C-H showed a sharp beak, O-H showed a weak beak, and C-O showed a very strong beak having a very strong structure between amino acid residues and synthesized proteins.

### 3.2. Dose Mortality Responses of S. litura to B. brongniartii Fe^0^NPs

**Figure 5:**
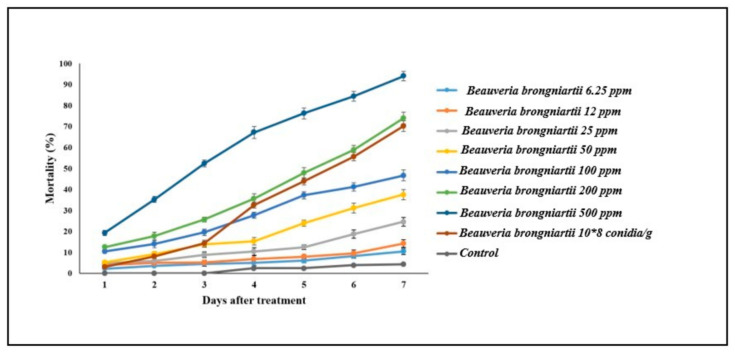
Percentage mortality of 2nd instar *Spodoptera litura* larvae in response to different treatments of *Beauveria brongniartii* Fe^0^NPs, *B. brongniartii,* and control at different time intervals. * Treatments (T1–T9) are outlined in Table 1. Error bars indicate the standard error of means.

Our results revealed that 100, 200, and 500 ppm concentrations of Fe^0^NPs induced higher *S. litura* mortality compared with *B.* *brongniartii* conidial suspension (1 × 10^8^ conidia/mL) alone until 3 days post-treatment. After this period 200 and 500 ppm concentrations of Fe^0^NPs caused higher *S. litura* mortality than *B.* *brongniartii* conidial suspension following 4 to 7 days of fungal treatment (Figure 5).

The LC_50_ values of Fe^0^NPs against *S. litura* after 7 days of fungal treatment was 58 ppm (Table 2). The LT_50_ values for 200 and 500 ppm concentrations of Fe^0^NPs against *S. litura* 7 days post-treatment were 5.10 and 2.29 days, respectively (Table 3).

### 3.3. Influence of B. brongniartii Fe^0^NPs on Feeding and Growth of Spodoptera litura

**Figure 6:**
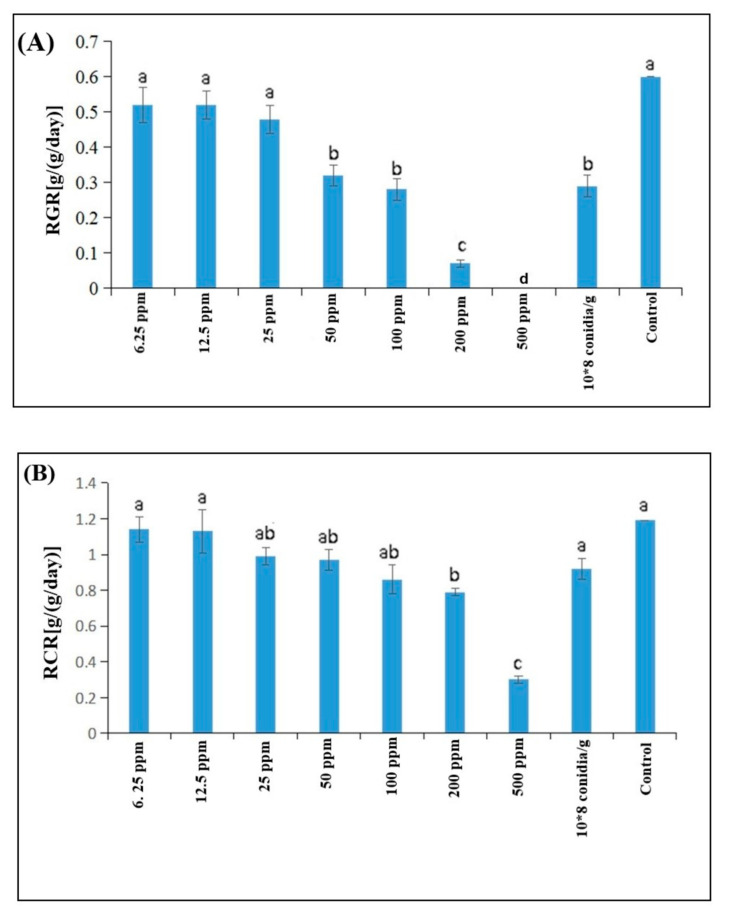

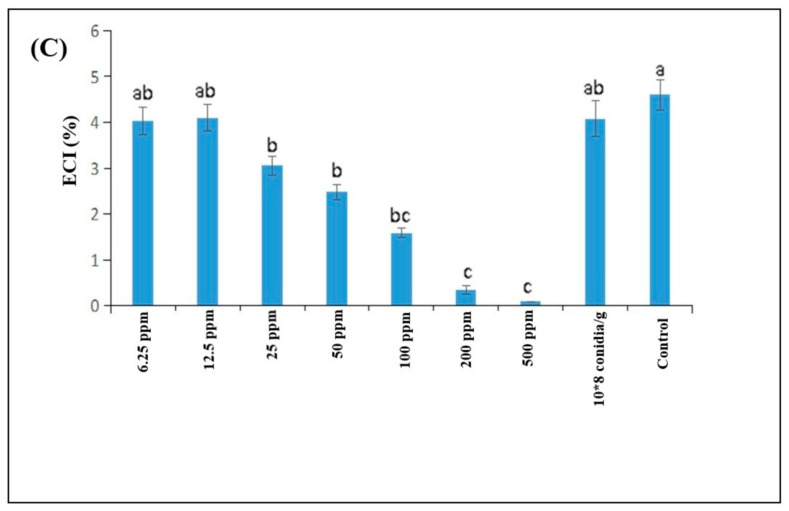
Feeding and growth parameters of 2nd instar *Spodoptera litura* larvae in response to different treatments of *Beauveria brongniartii* Fe^0^NPs, *B. brongniartii* conidia alone and control. (**A**) relative growth rate; (**B**) relative consumption rates and (**C**) percentage food conservation efficiency. Error bars indicate standard error of means. Different letters above columns indicate significant differences between means at a 5% level of significance.

The relative growth rate (RGR) of *S. litura* in response to *B. brongniartii* Fe^0^NPs application showed significant differences among different treatments at the end of the experimental period (F_8,18_ = 57.92; *p* < 0.001). The highest RGR of *S. litura* was observed for control whereas the lowest RGR was observed for Fe^0^NPs 500 ppm) (Figure 6A). The RGR observed for Fe^0^NPs 6.25 ppm, Fe^0^NPs 12.5 ppm, and Fe^0^NPs 25 ppm were similar to that of control whereas RGR values observed for Fe^0^NPs 50 ppm and Fe^0^NPs 100 ppm were similar to that of *B. brongniartii* conidial suspension 1 × 10^8^ conidia/g (Figure 6A).

Different treatments of *B. brongniartii* Fe^0^NPs significantly affected the relative consumption rates (RCR) of *S. litura* at the end of the experimental period (F_8,18_ = 49.54; *p* < 0.001). The highest RCR of *S. litura* was observed for control whereas the lowest RCR was observed for Fe^0^NPs 500 ppm (Figure 6B). The RGR observed for Fe^0^NPs 6.25 ppm, Fe^0^NPs 12.5 ppm, and *B. brongniartii* conidial suspension 1 × 10^8^ conidia/g were significantly similar to control whereas RGR values observed for Fe^0^NPs 25 ppm, Fe^0^NPs 50 ppm, and Fe^0^NPs 100 ppm were statistically similar (Figure 6B).

The index of food conservation efficiency (ECI) of *S. litura* in response to *B. brongniartii* Fe^0^NPs application differed significantly among different treatments at the end of the experimental period (F_8,18_ = 41.97; *p* < 0.001). The highest ECI values of *S. litura* were observed for control whereas the lowest ECI was observed for Fe^0^NPs 500 ppm. The ECI observed for Fe^0^NPs 200 ppm and Fe^0^NPs 500 ppm were similar to each other whereas ECI values observed for Fe^0^NPs 6.25 ppm and Fe^0^NPs 12.5 ppm were statistically similar to *B. brongniartii* conidial suspension 1 × 10^8^ conidia/g (Figure 6C).

### 3.4. Effects of B. brongniartii Fe^0^NPs on Activities of Detoxifying Enzymes

**Figure 7:**
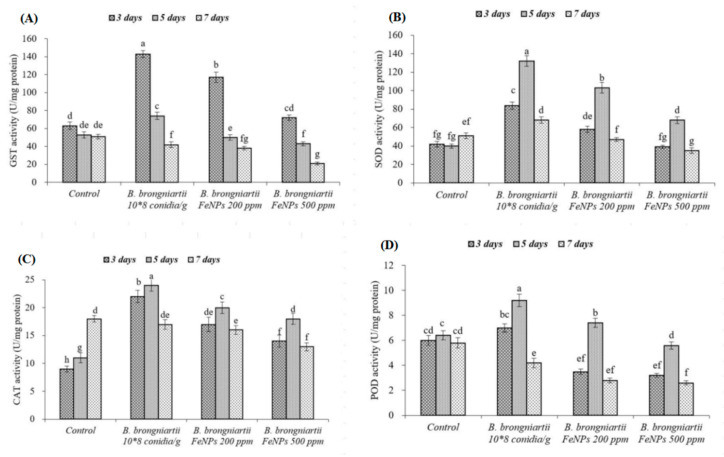
Detoxifying enzyme produced by 2nd instar *Spodoptera litura* larvae in response to different treatments of *Beauveria brongniartii* Fe^0^NPs, *B. brongniartii* and control after 3, 5, and 7 days of treatment. (**A**): glutathione-S-transferase (GST); (**B**) superoxide dismutase (SOD); (**C**) catalase (CAT); and (**D**) peroxidase (POD). Error bars indicate standard error of means. Different letters above columns indicate significant differences between means at a 5% level of significance.

The glutathione-S-transferase (GST) activities in *S. litura* fat bodies when treated with different concentrations of *B. brongniartii* Fe^0^NPs or *B. brongniartii* conidial suspension differed significantly from the control at different time intervals (F_11,19_ = 36.78; *p* < 0.001). (Figure 7A). The highest GST activity was observed 3 days post-treatment followed by a consistent decrease in enzyme activity in response to different treatments at 5 and 7 days of application (except for the control). The GST activities observed following Fe^0^NPs treatments were significantly lower than GST activities observed for *B. brongniartii* conidial suspension at 3, 5, and 7 days of treatment (Figure 7A).

The activities of different antioxidant enzymes (SOD, CAT, and POD) in *S. litura* fat bodies when treated with different concentrations of *B. brongniartii* Fe^0^NPs or *B. brongniartii *conidial** suspension also differed significantly from the control at 3, 5, and 7 days of treatment (SOD: F_11,19_ = 49.23, *p* < 0.001; CAT: F_11,19_ = 39.08, *p* < 0.001; POD: F_11,19_ = 28.21, *p* < 0.001). The enzyme activity values of antioxidant enzymes (SOD, CAT, and POD) showed an increasing-trends up to 5 days post-treatment followed then by a decrease in enzyme activity after 7 days of treatment. The antioxidant enzyme (SOD, CAT, and POD) activities observed for Fe^0^NPs treatments were significantly different from enzyme activities observed following the treatment of *B. brongniartii* conidial suspension at 3, 5, and 7 days of treatment (Figure 7B–D).

## 4. Discussion

The application of nanotechnology in combination with insect pathogens can be a promising biological alternative to synthetic chemicals for insect pest management [27]. Nanoparticles/nanomaterials-based formulations of insect pathogens have been recently tested as fungicides or insecticides for effective management of different insect pests and diseases [28,29]. During our previous studies, *I. fumosorosea-*Fe^0^NPs (100 ppm) application resulted in a 68% reduction in egg hatchability as well as 80–98% mortality of first, second, and third instar *B. tabaci* nymphs, respectively [13].

This study reports the use of *B. brongniartii* with Fe^0^NPs synthesis and their application for *S. litura* management. The treatment of *B. brongniartii* with Fe^0^ under dark conditions resulted in a change of filtrate color from light gray to dark gray showing the synthesis of Fe^0^NPs. The visualization of a characteristic peak between 400–450 nm in UV profile further confirmed the Fe^0^NPs synthesis. Our results are consistent with the findings of Wang et al. [13] who reported a similar change in the color of culture filtrate and a characteristic peak at 470 nm during bio-synthesis of *I. fumosorosea*-Fe^0^NPs. These findings are also in line with the results of Mukherjee et al. [30], Banu and Balasubramanian [10,11], and Amersan et al. [12] who also observed similar changes in culture filtrates during the synthesis of metal-based nanoparticles from different species of entomopathogenic fungi. The color change during the extracellular synthesis of Fe^0^NPs can be related/explained by two phenomena: (i) the excitation of surface plasmon vibration of nanoparticles [31]; (ii) metabolic utilization of nitrate through reduction of nitrate and ammonia during bio-reduction of metals [32]. The existence of Fe^0^ on *B. brongniartii* conidial surface was confirmed through scanning electron micrographs. The EDX spectroscopy showed two sharp peaks of iron at 6.5 and 7.1 KeV further confirming the absorption of metallic iron [33]. The XRD analysis showed characteristic peaks at 2*θ* values of 52.42° and 77.25°. The Fourier transformation infra-red spectrum also confirmed the presence of different biomolecules e.g., proteins which can be responsible for *B. brongniartii* Fe^0^NPs [11]. All these results are consistent with the findings of Wang et al. [13] who observed similar EDX, XRD, and FTIR patterns (with slight differences in peak values) during the extracellular synthesis of *I. fumosorosea* Fe^0^NPs.

During this study, 2nd instar *S. litura* larvae were treated with different concentrations of *B. brongniartii* Fe^0^NPs, resulting in larval mortality between 10% and 94% in response to different concentrations. The findings of this investigation are in line with previous studies on the toxicity of *I. fumosorosea* Fe^0^NPs against *B. tabaci* by Wang et al. [13] who reported 23–98% mortality of 2nd instar *B. tabaci* nymphs in response to different concentrations of *I. fumosorosea* Fe^0^NPs. The indices probit analysis such as median lethal concentration (LC_50_) and median lethal time (LT_50_) are commonly used parameters to gauge the efficacy of any pest control agent. The median lethal concentration (LC_50_) values of *B. brongniartii* Fe^0^NPs against 2nd instar *S. litura* larvae were 58 ppm after 7 days of treatment. Wang et al. [15] reported the median lethal concentration (LC_50_) value of 19.17 for *I. fumosorosea* Fe^0^NPs against 2nd instar *B. tabaci* nymphs. Banu and Balasubramanian [10] found the LC_50_ value of 0.79 ppm for *B. bassiana* AgNPs against 2nd instar larvae of *Aedes aegypti*. The median lethal time (LT_50_) values for 200 and 500 ppm concentrations of *B. brongniartii* Fe^0^NPs against 2nd instar *S. litura* larvae were 5.10 and 2.29 days, respectively. The LT_50_ values of this study were a little higher than the findings of Wang et al. [13] who reported an LT_50_ value of 3.15 days for 100 ppm concentration of *I. fumosorosea* Fe^0^NPs against 2nd instar *B. tabaci* nymphs following 7 days of fungal treatment.

The nutrition analysis to study the effects of *B. brongniartii* Fe^0^NPs on feeding and growth parameters of *S. litura* showed a significant reduction in RCR, RGR, and ECI values compared with the control following the application of different *B. brongniartii* Fe^0^NPs concentrations. Although *B. brongniartii* conidial suspension also caused a reduction in RCR, RGR, and ECI values compared with the control, the changes were more prominent with higher concentrations of *B. brongniartii* Fe^0^NPs. These changes in RCR, RGR, and ECI values can be explained by different phenomenon or hypothesis such as: (1) enhanced degradation of the insect cuticle or delayed ecdysis in response to *B. brongniartii* Fe^0^NPs due to a combined action of extracellular hydrolytic enzymes produced by fungi known for their effect on an insect cuticle [34,35] and Fe^0^ particles known to degrade hydrocarbons in nature or alkanes present in an insect cuticle [13,36]; (2) possible degradation of the insect gut peritrophic membranes by chitinase produced by the fungi [20,37,38] and also the degradation of the peritrophic membrane being further enhanced by the addition of Fe^0^ which is known for its ability to degrade hydrocarbons and aromatic structures in nature [39]; (3) decreased efficiency to convert the consumed food into a growth and energy source through the possible diversion of energy from growth to the detoxification process [19,20].

In insects, the detoxification of foreign pathogens or chemicals is accomplished by different detoxifying and antioxidant enzymes [16,40,41]. Glutathione-S-transferase (GST) is the main enzyme involved in the detoxification of insecticides or pathogens in insect bodies [40]. Our findings demonstrated an increase in GSTs activity during the initial 72 h followed then by a reduction in enzyme activity throughout the experimental period in response to the application of *B. brongniartii* or different concentrations of *B. brongniartii* Fe^0^NPs. This increase in enzyme activity during the initial infection period is a clear indication of the possible involvement of GSTs in the insect detoxification mechanism. The reduction in enzyme activity during 72 h post-infection can make the target host more susceptible to infection leading to a metabolic imbalance and insect death [41]. The antioxidant enzymes (SOD, CAT, and POD) are well known for their role in detoxifying destructive antioxidant species [42]. In our study, the activities of antioxidant enzymes (SOD, CAT, and POD) increased (until 5 days post-treatment) followed then by a decrease compared with the control when treated with *B. brongniartii* or different concentrations of *B. brongniartii* Fe^0^NPs. The antioxidant enzyme (SOD, CAT, and POD) activities in response to *B. brongniartii* Fe^0^NPs were significantly lower than *B. brongniartii* after 3, 5, and 7 days of treatment. The reduced activities of different antioxidant enzymes (SOD, CAT, and POD) during the later experimental period can lead to a lower elimination of reactive oxygen species which in turn can denature different bio-molecules within an insect body. The denaturation of bio-molecules stops all cellular processes which ultimately leads to the death of the insect [43].

## 5. Conclusions

In summary, this study characterized the bio-insecticidal activity of *B. brongniartii* Fe^0^NPs against *S. litura* but also provides information on the effects of *B. brongniartii* Fe^0^NPs on insect growth parameters as well as the detoxifying mechanism within *S. litura*. All the above findings suggest that *B. brongniartii* Fe^0^NPs can potentially be used within *S. litura* management programs. However, further work is required to determine the efficacy and persistence of *B. brongniartii* Fe^0^NPs under field conditions.

## Figures and Tables

**Table 1:** Details of different *Beauveria brongniartii* Fe^0^ nanoparticles and *B. brongniartii* treatments used in the bio-efficacy studies.

Treatment	Treatment Description	Concentration
**T1**	*B. brongniartii* Fe^0^NPs	6.25 ppm of diet
**T2**	*B. brongniartii* Fe^0^NPs	12.5 ppm of diet
**T3**	*B. brongniartii* Fe^0^NPs	25 ppm of diet
**T4**	*B. brongniartii* Fe^0^NPs	50 ppm of diet
**T5**	*B. brongniartii* Fe^0^NPs	100 ppm of diett
**T6**	*B. brongniartii* Fe^0^NPs	200 ppm of diet
**T7**	*B. brongniartii* Fe^0^NPs	500 ppm of diet
**T8**	*B. brongniartii* conidial suspension	10^8^ conidia/g of diet
**T9**	Control (ddH_2_O)	0

**Table 2:** Median lethal concentration (LC_50_) values of *Beauveria brongniartii* Fe^0^NPs against *Spodoptera litura* estimated by probit regression.

Treatment Time (Days)	LC_50_ (ppm)	95% Fiducial Limit	Slope ± S.E	χ^2^ (df = 4)	*p*
7	58	36–95	1.63 ± 0.13	3.92	0.870

**Table 3:** Median lethal time (LT_50_) values of *Beauveria brongniartii* Fe^0^NPs nanoparticles against *Spodoptera litura* estimated by probit regression.

Concentration (ppm)	LT50 (Days)	95% Fiducial Limit	Slope	χ^2^ (df = 4)	*p*
200	5.10	3.72–6.99	2.32 ± 0.24	4.77	0.869
500	2.29	1.72–3.06	2.63 ± 0.19	3.92	0.964

## Supplementary Materials

The following are available online at https://www.mdpi.com/2075-4450/11/12/895/s1, Figure S1: Changes in colour of culture filtrate during extracellular synthesis of *Beauveria brongniartii* Fe^0^NPs (A) *B. brongniartii* conidial filtrate; (B) 1 mM Fe^0^; and (C) *B. brongniartii* Fe^0^NPs, Figure S2: Energy dispersive X-ray spectroscopy profile of *Beauveria brongniartii* Fe^0^NPs.
