# Supplementary material for: Toxicity and Biological Effects of Beauveria brongniartii Fe0 Nanoparticles against Spodoptera litura (Fabricius)
Source: Insects. 2020 Dec 21;11(12):895. doi: 10.3390/insects11120895 (PMC7767332; doi:10.3390/insects11120895)
Supplement: Supplementary file 1 [file insects-11-00895-s001.pdf]

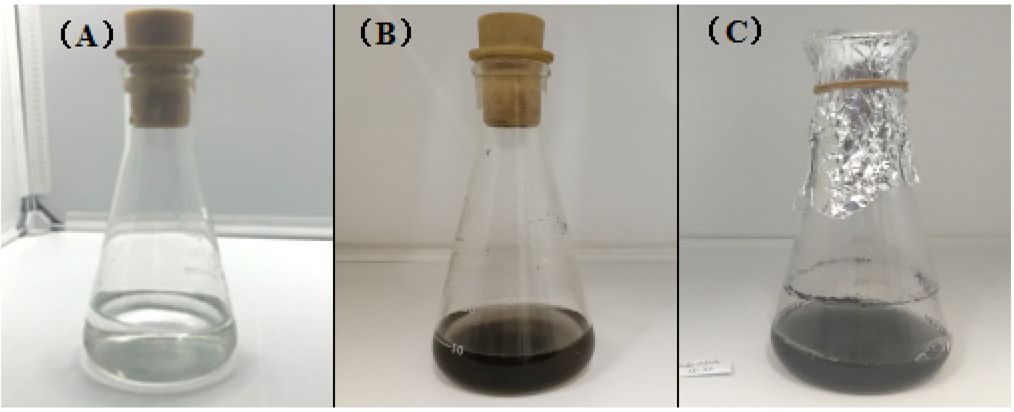

Supplementary Figure S1. Changes in colour of culture filtrate during extracellular synthesis of *Beauveria brongniartii* Fe^0NPs (A) *B. brongniartii* conidial filtrate; (B) 1 mM Fe^0; and (C) *B. brongniartii* Fe^0NPs.

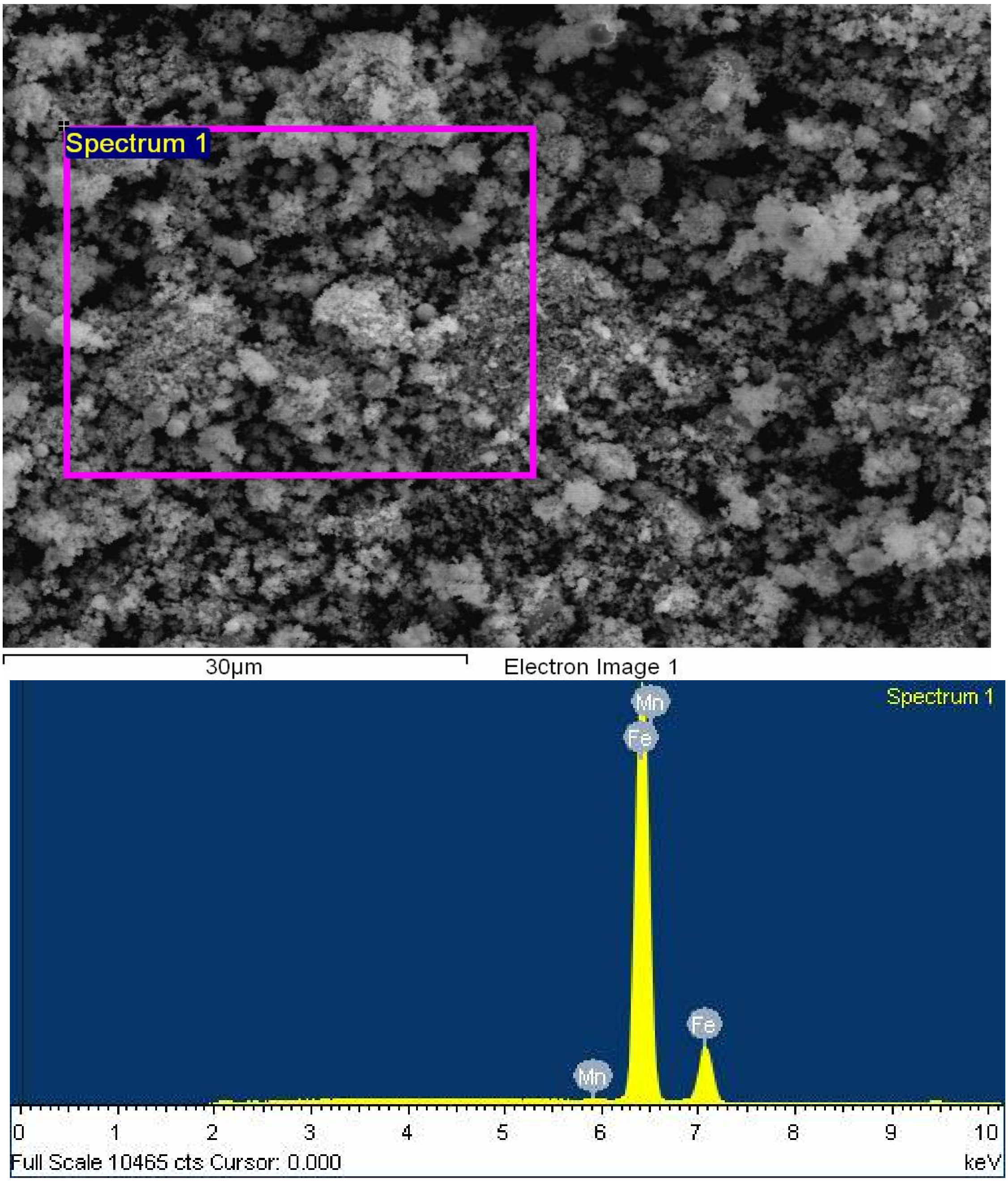

Supplementary Figure S2. Energy dispersive X-ray spectroscopy profile of *Beauveria brongniartii* Fe^0NPs.
